# First autochthonous human West Nile virus infections in the Netherlands, July to August 2020

**DOI:** 10.2807/1560-7917.ES.2020.25.46.2001904

**Published:** 2020-11-19

**Authors:** Danique RM Vlaskamp, Steven FT Thijsen, Johan Reimerink, Pieter Hilkens, Willem H Bouvy, Sabine E Bantjes, Bart JM Vlaminckx, Hans Zaaijer, Hans HTC van den Kerkhof, Stijn FH Raven, Chantal BEM Reusken

**Affiliations:** 1 Department of Neurology, St. Antonius hospital, Nieuwegein, the Netherlands; 2 These authors contributed equally; 3 Department of Medical Microbiology and Immunology, Diakonessenhuis Hospital, Utrecht, the Netherlands; 4 Centre for Infectious Disease Control, National Institute for Public Health and the Environment, Bilthoven, the Netherlands; 5 Department of Neurology, Diakonessenhuis Hospital, Utrecht, the Netherlands; 6 Department of Medical Microbiology and Immunology, St. Antonius Hospital, Nieuwegein, the Netherlands; 7 Sanquin Blood Supply Foundation and Amsterdam University Medical Centre, Amsterdam, the Netherlands; 8 Department of Infectious Diseases, Public Health Service region Utrecht, Utrecht, the Netherlands

**Keywords:** West Nile virus, the Netherlands, zoonoses, blood safety, meningitis, WNND, WNF

## Abstract

In October 2020, the first case of autochthonous West Nile virus neuroinvasive disease was diagnosed in the Netherlands with a presumed infection in the last week of August. Investigations revealed five more cases of local West Nile virus (WNV) infection. The cases resided in a region where WNV was detected in a bird and mosquitoes in August 2020. Molecular analysis was successful for two cases and identified the presence of WNV lineage 2.

In October 2020, a case of autochthonous West Nile virus neuroinvasive disease (WNND) was diagnosed in the Netherlands with a presumed date of infection in the last week of August. Additional epidemiological investigations and retrospective serological analyses of cerebrospinal fluid (CSF) from patients with neurological disease of unknown but suspected viral aetiology sampled between August and September 2020, revealed five more cases of West Nile virus (WNV) infection. Three of them had WNND and two had West Nile fever (WNF) [1,2]. The cases were infected between July and August 2020, and all of them resided in a region where WNV lineage 2 was detected in a bird and mosquitoes in August 2020 [3]. Molecular analysis was successful for two cases showing the presence of WNV lineage 2.

## Cases 1 and 2

At the end of August 2020, a person in their mid-70s (Case 1) developed a generalised rash, as did their spouse (Case 2, also in their mid-70s) one day later. Both cases’ rashes disappeared after a few days, but Case 1 developed a high fever with severe malaise on day 5 after symptom onset. Case 1 was weak, unable to walk and barely able to speak. From day 6 onwards, Case 1 further deteriorated, with a headache, vomiting, urinary incontinence, confusion and slowness of thought and movements, and was admitted to Hospital A (day 8). At admission, Case 1 was disoriented in time and place and had cognitive impairment and walking apraxia. Neurological examination revealed no focal neurological deficits. Clinically, Case 1 was diagnosed with an encephalitis.

Laboratory results, chest X-ray and urine investigation showed no signs of active infection (Supplementary Table). CSF investigation revealed mild pleocytosis with mononuclear cells and a slightly elevated protein level. Serological and CSF investigations excluded a range of infectious diseases, including infection with tick-borne encephalitis virus (TBEV). Magnetic resonance imaging revealed no abnormalities. Clinically, Case 1’s cognition slowly improved, but at discharge (day 40 post symptom onset) the patient was still unable to walk and was referred to a rehabilitation centre.

In early October, continued laboratory investigations at the National Institute for Public Health and the Environment (RIVM) confirmed WNV infection as the causative agent for WNND (Table; [1]). Three consecutive serum samples were positive for WNV IgM and IgG in an ELISA (Euroimmun, Lübeck, Germany); they were also positive for WNV IgG, but not Usutu virus (USUV) or TBEV IgG, in a multiplex flavivirus protein-array [4]. Comparative plaque reduction neutralisation test (PRNT)_50_ for WNV and USUV [5] confirmed the presence of WNV neutralising capacity, but not USUV. Reverse-transcription (RT)-PCR on the cell-pellet of whole blood taken on day 42 was positive (cycle threshold (Ct) value: 33) for WNV lineage 2 but not WNV lineage 1 (in house [6-8]). Amplicon-based whole genome sequencing identified two WNV lineage 2 genome fragments (417 and 415 bp) identical to a WNV sequence found in a Dutch mosquito pool (GenBank MW228499 and MW228500; GenBank MW036633 [3]).

**Table:** Sampling schedule and flavivirus diagnostic results, autochthonous human cases of West Nile virus infection, the Netherlands, August–October 2020 (n = 6)

Sample type	Case 1			Case 2	Case 3		Case 4	Case 5		Case 6	
	Serum	Serum	Serum/ whole blood	Serum/ whole blood	CSF	Serum/ whole blood	Serum/ whole blood	CSF	Serum/ whole blood	CSF	Serum/ whole blood
Sampling month and days post symptom onset	Sep 19 dpo	Sep 29 dpo	Oct 43 dpo	Oct 46 dpo	Aug 16 dpo	Oct 68 dpo	Oct 68 dpo	Sep 7 dpo	Oct 65 dpo	Aug 25 dpo	Oct 98 dpo
WNV ELISA IgM/IgG^a^	2.47/1.71	2.72/1.91	2.50/3.08	4.03/5.47	6.83/Neg	Neg/4.83	2.72/4.43	1.89/5.21	Neg/5.02	3.83/Neg	Neg/Neg
WNV RT-PCR Ln 2/Ln 1	NT	NT	Pos Ct 33	Pos Ct 34	NT	Neg	Neg	NT	Neg	NT	Neg
WNV PRNT_50_ /Usutu PRNT_50_^b^	1,024/NT	1,024/NT	512/Neg	1,024/Neg	NT	512/NT	256/NT	NT	256/NT	NT	NT
Multiplex flavivirus NS1 micro-array: WNV Ln 2/Ln 1^c^	108/Neg	121/63	258/86	774/307	NT	> 1,280/927	402/270	NT	> 1,280/ > 1,280	NT	63/27
USUV	Neg	Neg	Neg	84	NT	262	79	NT	331	NT	NT
TBEV	Neg	Neg	Neg	Neg	NT	53	Neg	NT	72	NT	NT

Ct: PCR cycle threshold; CSF: cerebrospinal fluid; dpo: days post onset of symptoms; ELISA: enzyme-linked immunosorbent assay; Ln 1: lineage 1; Ln 2: lineage 2; Neg: negative; NS1: flavivirus non-structural protein 1; NT: not tested; Pos: positive; PRNT: plaque reduction neutralisation assay; TBEV: tick-borne encephalitis virus; USUV: Usutu virus; WNV: West Nile virus.

^a^ Optical density ratios.

^b^ Neutralising antibody titres.

^c^ IgG titres (cut-off titre is 10).

Reactivity with WNV Ln 1, USUV and/or TBEV NS1 is cross-reactivity of WNV Ln 2 NS1 directed IgG [4].

Following Case 1’s diagnosis, whole blood was collected from Case 2 on day 45 post rash onset. ELISA, protein micro-array, PRNT_50_ and RT-PCR confirmed WNF caused by a WNV lineage 2 strain [1].

Both cases had no relevant travel history and had no recollection of mosquito bites in the weeks before onset of symptoms; however, they lived in the region where WNV was found in a bird and mosquitoes [3].

## Active case finding

To rapidly investigate the extent of WNV circulation and human infection in the affected region, two regional hospitals were asked to submit CSF of patients who were hospitalised in August and September 2020 with unexplained neurological disease of suspected viral origin to RIVM to analyse for the presence of WNV IgM and IgG (ELISA).

Analysis of CSF from 64 patients of Hospital B for the presence of WNV IgM and IgG identified a second patient with WNND (Case 3). Further, analyses of CSF of 37 patients with neurological disease in Hospital A confirmed two additional WNND patients (Cases 5 and 6). For all cases, laboratory investigations corroborated the diagnoses (Table; Supplementary Table).

## Cases 3 to 6

Case 3, an otherwise healthy person in their mid-30s, visited their general physician in mid-August for severe headache that had started 8 days earlier, as well as muscle ache and skin rash that had started 1 day prior. Doxycycline was prescribed for presumed leptospirosis. The case was referred to the emergency ward 2 days later with worsening symptoms (fever of 39.5 °C, vertigo, neck pain, nausea and vomiting). Neurological examination was normal (no neck stiffness) except for a first-degree horizontal nystagmus. Laboratory results showed normal infection parameters (Supplementary Table). A lumbar puncture showed mild pleocytosis of mostly mononuclear cells (93%) and a mildly elevated protein. The case was treated with acyclovir, ceftriaxone and trimethoprim/sulfamethoxazole. Therapy was stalled when test results for *Leptospira* spp., herpes simplex virus, varicella zoster virus, severe acute respiratory syndrome coronavirus 2 and *Borrelia* spp. were negative. Symptoms improved rapidly and the case was discharged on day 15 after the first symptom onset. Case 3’s outpatient visit 2 months later revealed mild persisting fatigue and irritability.

Epidemiological investigation for Case 3 revealed that their partner (Case 4), in their early 30s, had experienced a rash, retro-orbital pain and malaise in the same period, but without progression to neurological disease. Laboratory investigation confirmed that Case 4 had a recent WNV infection (Table).

Cases 3 and 4 had not travelled outside the Netherlands in the 2 weeks before the onset of symptoms. Both lived in the region where the WNV-infected bird and mosquitoes were found [3] and experienced mosquito bites in the week before onset of symptoms.

Case 5 was in their late 60s and resided in the same municipality as Cases 1 and 2. Case 5 experienced illness at the end of August. Case 6 was an unrelated case in their mid-40s that lived ca 20 km from the location where the WNV-positive bird and mosquitoes were found. Case 6’s first day of illness was at the end of July 2020. They received immunosuppressive therapy since early October 2020, which might explain the absence of ELISA-detectable WNV IgG and IgM in serum collected on day 98 after symptom onset. Both cases had not travelled outside the Netherlands and had no recollection of mosquito bites in the weeks before disease onset.

## Ethical statement

The diagnostics performed herein were either part of routine clinical microbiology practice to establish the cause of illness of unexplained cases in medical care or part of public health usual practice.

## Discussion

The identification of the first six clinical cases of autochthonous human WNV infection in the Netherlands confirms this mosquito-borne virus’ potential to expand its geographical distribution northward. Since 2018, WNV has established a presence in Europe as far north as Germany [9], though areas in south-eastern Europe still report the highest number of human cases. As at 12 November 2020, 315 WNV cases from eight countries have been notified to the European Centre for Disease Prevention and Control, with the majority of cases from Greece (n = 143), Italy (n = 66) and Spain (n = 77) [10].

Between August and September 2020, local presence of WNV in the Netherlands was observed for the first time with the detection of WNV RNA in a common whitethroat and in two *Culex* spp. mosquito pools in the municipality of Utrecht [3]. RIVM announced this first observation to the public on 16 September 2020 [11] and details were shared with infectious disease professionals on 17 September 2020 using a weekly national alerting system [12]. The first identification of autochthonous cases of WNND in the Netherlands underlines the importance of a One Health approach to surveillance for arboviruses and rapid risk communication to clinical professionals and the general public.

The clinical and epidemiological investigations described herein focused on a specific region and were triggered by the possible enzootic presence of WNV. Following the diagnosis of the first WNND case, local syndrome-based investigations identified three more cases of WNND (Cases 3, 5 and 6) and two cases of WNF (Cases 2 and 4). Research has shown that for each case of WNND detected, on average 250 other mild or asymptomatic infections occur [13,14]; based on the four known WNND cases in the region described here, it can be expected that hundreds of people were infected in this area during the summer months. The extent of WNV presence in Utrecht and adjacent regions will be investigated through (i) retrospective screening of CSF of unexplained neurological patients and (ii) regional seroprevalence studies in humans, as well as (iii) entomological and (iv) veterinary research.

As WNV can be transmitted through blood transfusion, it is important to determine the geographical extent of circulation. In line with European Union directives, safety measures were applied to blood donations from donors residing or donating in the affected areas [15]. In October 2020, a seventh case became infected in another region in the Netherlands (Arnhem) and blood safety measures were expanded accordingly [16].

Although it remains to be seen whether WNV will overwinter and maintain an enzootic presence in the Netherlands, as can be expected based on spatial risk analysis and in analogy with the situation in Germany [9,17], further multidisciplinary surveillance and communication strategies will be developed in the coming months to monitor the extent of spread and increase awareness of the disease among clinicians and the general public.

## Supplementary Data

637000 Supplementary Table
