# Supplementary material for: First autochthonous human West Nile virus infections in the Netherlands, July to August 2020
Source: Euro Surveill. 2020 Nov 19;25(46):2001904. doi: 10.2807/1560-7917.ES.2020.25.46.2001904 (PMC7678035; doi:10.2807/1560-7917.ES.2020.25.46.2001904)
Supplement: Supplementary Table [file 2001904_REUSKEN_FINALsupplement.pdf]

Supplementary table “Results of additional laboratory investigation” to Vlaskamp *et al*. First autochthonous human West Nile virus infections in the Netherlands, July-August 2020.

This supplementary material is hosted by Eurosurveillance as supporting information alongside the article “First autochthonous human West Nile virus infections in the Netherlands, July to August 2020” on behalf of the authors who remain responsible for the accuracy and appropriateness of the content. The same standards for ethics, copyright, attributions and permissions as for the article apply. Supplements are not edited by *Eurosurveillance* and the journal is not responsible for the maintenance of any links or email addresses provided therein.

Supplementary table. Results of additional laboratory investigation.

Investigation	Test	Case 1 Result	Case 1 Days post onset symptoms	Case 3 Result	Case 3 Days post onset symptoms	Case 5 Result	Case 5 Days post onset symptoms	Case 6 Result	Case 6 Days post onset symptoms
**Laboratory tests**	Hb (mmol/L)	8.7	8	9.3	10	9.7	4	6.8	1
	Leucocytes (*10^9/L mmol/L)	11.8	8	8.8	10	12.6	4	27.2	1
	Trombocytes (*10^9/L mmol/L)	228	8	154	10	183	4	545	1
	CRP (mg/L)	37	8	<1	10	<1	4	17	1
	Kreatinin (µmol/L)	nt	na	88	10	nt	na	nt	na
	eGFR (MDRD4; ml/min/1.73 m2	>90	8	nt	na	48	4	nt	na
	Albumine (g/L)	39.6	8	nt	na	43.8	4	nt	na
	Glucose (mmol/L)	5.9	8	nt	na	16.5	4	nt	na
	ALAT (U/L)	27	8	nt	na	30	4	nt	na
	ALAT (U/L)	23	8	nt	na	31	4	nt	na
	Gamma-GT (U/L)	44	8	nt	na	45	4	nt	na
	AF (U/L)	68	8	nt	na	47	4	nt	na
	Billirubine (mmol/L)	9	8	nt	na	29	4	nt	na
	Ferritin (ug/L)	577	8	nt	na	nt	na	nt	na
	CK (U/L)	46	8	nt	na	nt	na	nt	na
**Bloodculture**		No growth	8	No growth	10	No growth	4	nt	na
**Urine**	Leucocytes (/ul)	<1	8	nt	na	2	4	nt	na
	Nitrite test	Positive	8	nt	na	Negative	4	nt	na
	Urine culture	Negative	8	nt	na	nt	na	nt	na
**Serum**	CMV IgG and IgM	Negative	18	Negative	10	nt	na	nt	na
	EBV IgM and IgG	Negative	18	past infection	10	nt	na	nt	na
	Toxoplasmosis IgM and IgG	Negative	18	nt	na	nt	na	nt	na
	Borrelia IgM and IgG	Negative	18 and 27	Negative	10	nt	na	nt	na
	T. pallidum antibodies	Negative	18	nt	na	nt	na	nt	na
	HIV antibodies	Negative	18	nt	na	nt	na	nt	na
	Ticke Borne IgM and IgG	Negative	18	nt	na	nt	na	nt	na
	Neuronal antibodies*	Negative	18	nt	na	nt	na	nt	na
	Herpes simplex 1 and 2 PCR	Negative	25	nt	na	nt	na	nt	na
	Leptospirosis PCR/IgM	nt	na	Negative	10	nt	na	nt	na
**CSF**	Leucocytes (/µl)$	22	13	117	11	33	7	nt	na
	Erytrocytes (/µl)	<1000	13	300	11	<1000	7	nt	na
	Protein (mg/L)	849	13	860	11	668	7	nt	na
	Glucose (mmol/L)	3.6	13	3.4	11	4.2	7	nt	na
	Culture	No growth	13 and 18	No growth	11	nt	na	nt	na
	CMV PCR	nt	na	nt	na	Negative	7	nt	na
	EBV PCR	nt	na	nt	na	Negative	7	nt	na
	Entero- and parecho PCR	Negative	13	Negative	11	Negative	7	nt	na
	Herpes simplex 1 and 2 PCR	Negative	13	Negative	11	Negative	7	nt	na
	Varicella zoster PCR	Negative	13	Negative	11	nt	na	nt	na
	Mycobacterium PCR	Negative	18	Negative	11	nt	na	nt	na
	Borrelia PCR	Negative	18	nt	na	nt	na	nt	na
	Borrelia IgG/IgM index liquor/seru	<1.5	18	nt	na	nt	na	nt	na
	Neuronal antibodies **	Negative	18	nt	na	nt	na	nt	na
**Fluid**	Subcutaneous fluid pocket connected with cerebral spinal fluid	nt	na	nt	na	nt	na	Pseudomonas^	Unknown

*Neuronal antibodies that were tested in serum included anti-VGKC, anti-amphiphysine, anti-CV2, anti-PNMA2, anti-Ri, anti-Yo, anti-Hu, anti-recoverine, anti-SOX1, anti-zic4, ant-GAD65 and ant-Tr (DNER). ** Neuronal antibodies that were tested in liquor were anti-NMDA-R, anti-AMPA-R and anti-GABA-R. $93% mononuclear. ^pseudomonas infection was described for this case after liquor was send in for WNV IgM analysis for unexplained viral neurological disease. nt = not tested; na = not applicable.
